# Development of Surveillance Robots Based on Face Recognition Using High-Order Statistical Features and Evidence Theory

**DOI:** 10.3390/jimaging12030107

**Published:** 2026-02-28

**Authors:** Slim Ben Chaabane, Rafika Harrabi, Anas Bushnag, Hassene Seddik

**Affiliations:** 1 Computer Engineering Department, Faculty of Computers and Information Technology, University of Tabuk, Tabuk 47512, Saudi Arabia; rharrabi@ut.edu.sa (R.H.); abushnag@ut.edu.sa (A.B.); 2 Laboratoire de Robotique Intelligente, Fiabilité Et Traitement du Signal Image (RIFTSI), Ecole Nationale Supérieure d’Ingénieurs de Tunis (ENSIT)-Université de Tunis, Tunis 1008, Tunisia; seddikhassne@gmail.com

**Keywords:** recognition, Raspberry PI, robot, surveillance, face recognition, classification, membership degree, evidence theory, mass function

## Abstract

The recent advancements in technologies such as artificial intelligence (AI), computer vision (CV), and Internet of Things (IoT) have significantly extended various fields, particularly in surveillance systems. These innovations enable real-time facial recognition processing, enhancing security and ensuring safety. However, mobile robots are commonly employed in surveillance systems to handle risky tasks that are beyond human capability. In this paper, we present a prototype of a cost-effective mobile surveillance robot built on the Raspberry PI 4, designed for integration into various industrial environments. This smart robot detects intruders using IoT and face recognition technology. The proposed system is equipped with a passive infrared (PIR) sensor and a camera for capturing live-streaming video and photos, which are sent to the control room through IoT technology. Additionally, the system uses face recognition algorithms to differentiate between company staff and potential intruders. The face recognition method combines high-order statistical features and evidence theory to improve facial recognition accuracy and robustness. High-order statistical features are used to capture complex patterns in facial images, enhancing discrimination between individuals. Evidence theory is employed to integrate multiple information sources, allowing for better decision-making under uncertainty. This approach effectively addresses challenges such as variations in lighting, facial expressions, and occlusions, resulting in a more reliable and accurate face recognition system. When the system detects an unfamiliar individual, it sends out alert notifications and emails to the control room with the captured picture using IoT. A web interface has also been set up to control the robot from a distance through Wi-Fi connection. The proposed face recognition method is evaluated, and a comparative analysis with existing techniques is conducted. Experimental results with 400 test images of 40 individuals demonstrate the effectiveness of combining various attribute images in improving human face recognition performance. Experimental results indicate that the algorithm can identify human faces with an accuracy of 98.63%.

## 1. Introduction

Surveillance has become really important for keeping our lives and belongings safe in various areas over the past few years. As security threats have gotten more complicated—think theft, vandalism, terrorism, and cybercrime—needing smarter surveillance systems has grown [1,2]. These tools are now being used more often in public places, private properties, industrial sites, and government buildings to help ensure our safety.

The integration of mobile robots into surveillance systems [3,4] provides a highly flexible and cost-effective solution to enhance safety across various sectors, including industrial applications, military operations, and home automation. These robots can effectively replace humans in hazardous environments or risky manufacturing processes, decreasing the necessity for human involvement in unsafe situations. Their capacity for autonomous navigation and real-time task execution renders them an essential resource for enhancing security and safety while simultaneously reducing operational costs.

Intelligent security robots [5,6] are equipped with cameras and a variety of sensors that allow them to continuously monitor an area with minimal human oversight. They can detect intrusions or issues and alert surveillance personnel in real-time with high reliability. Subsequently, surveillance mobile robots have become a significant focus of research, given their potential to enhance security and operational efficiency.

Surveillance technologies have significantly advanced with the integration of modern innovations such as artificial intelligence (AI), computer vision (CV), and the Internet of Things (IoT). These technologies enable real-time monitoring, anomaly detection, and automated responses, making surveillance systems more efficient and reliable. For instance, face recognition, behavior analysis, and motion detection are now commonplace in modern surveillance, allowing for the early detection of potential threats.

Recently, the need for secure identification methods has become increasingly crucial. Traditional methods such as passwords and PIN codes are no longer enough to protect sensitive information and secure access to restricted areas [7,8,9]. This has led to the rise in biometric technology, which uses unique physical or behavioral characteristics to verify an individual’s identity. In recent years, many security structures have started implementing biometric systems based on iris, fingerprint [8], voice, and face recognition [10].

Due to the diverse structures and features present in the human face, it has emerged in recent years as one of the most widely employed biometric authentication systems in various applications and domains [7,8,9]. A robust face recognition system [10] is developed using three fundamental steps: face detection, feature extraction, and face matching/classification. The process of face detection begins by locating and identifying the human face. The feature extraction step is a critical aspect of the recognition process since it is responsible for extracting feature vectors for any human face identified in the initial stage. Moreover, successful feature extraction plays a pivotal role in determining the success of subsequent steps. Finally, the face recognition process conducts matching or classification of image features according to predefined criteria and is then compared against all template face databases to establish the identity of a human face.

**Figure 1:**
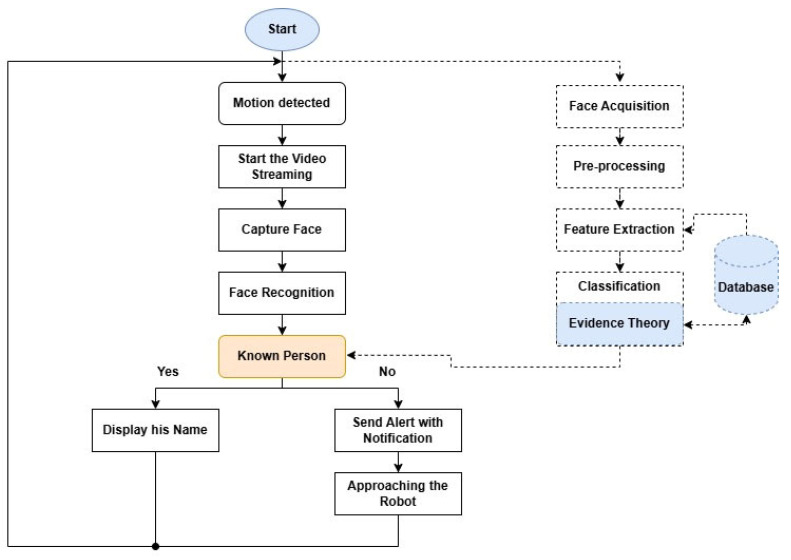
Unified System architecture and workflow.

Figure 1 illustrates the overall architecture of the proposed intelligent surveillance system, combining the face recognition framework with the operational workflow of the mobile robot. The figure provides a unified view of the main processing stages, including face detection, feature extraction using high-order statistical features, evidence theory-based classification, and IoT-based alert and control mechanisms. By integrating both the system structure and operational flow into a single diagram, the proposed architecture is presented in a clearer and more concise manner, avoiding redundancy and improving the understanding of the interactions between hardware components, software modules, and decision-making processes.

Figure 1 uses different line styles and colors to illustrate the system structure and functionality clearly. Solid lines represent the main operational workflow, showing the sequential execution of processes from motion detection to the final system response. In contrast, dotted lines indicate supporting or auxiliary interactions, including data exchange and logical connections with internal processing stages and the database. Gray blocks denote the standard processing steps carried out by the system, while the yellow block (Known Person) represents the main decision node that determines whether the detected individual is recognized or unknown. The blue block (Evidence Theory) highlights the decision fusion module used to improve classification reliability. The cylindrical shape represents the database, which stores information used for feature comparison and identity verification. Additionally, dashed-outline blocks correspond to internal algorithmic stages or backend processes operating within the face recognition subsystem.

The primary goal of this research is to design and implement a cost-effective intelligent mobile surveillance robot capable of performing real-time intruder detection and identification in industrial and security-sensitive environments. To achieve this goal, the study proposes a hybrid face recognition framework that combines high-order statistical feature extraction with Dempster–Shafer evidence theory, integrated within an IoT-based robotic surveillance platform using Raspberry Pi 4. The proposed approach aims to improve recognition accuracy and robustness under challenging conditions such as illumination variations, facial expressions, and partial occlusions, while ensuring real-time operation and remote monitoring capabilities.

Face recognition is a technique used to authenticate or identify individuals by automatically recognizing them based on their facial characteristics. It involves a computer program that can automatically detect or confirm one or more individuals from a digital image, or a video frame extracted from a video source. In verification situations, the similarity between two facial images is evaluated, and a decision is made regarding whether they match or not. The process of identifying the identity of an unknown facial image from a database of recognized faces is referred to as face recognition. In this scenario, the system computes the similarity between a particular facial image and all facial images stored in a large database, returning the best match as the presumed identity of the person.

In this context, many authors have examined the issue of face recognition by employing diverse techniques [5,6], and many authors and researchers have extensively discussed the challenges and advantages of these hybrid approaches in the context of face recognition [11]. They have explored how combining different techniques can enhance performance under various conditions such as varying lighting conditions, pose variations, occlusions, and facial expressions [7,8,9].

The Internet of Things (IoT) is a recent advancement in surveillance technology designed to bolster security and provide personalized protection. This innovative approach involves integrating a wide range of physical devices—such as cameras, sensors, and alarms—into a network connected to the internet. These interconnected devices can communicate with one another and with centralized systems to collect, analyze, and share data in real-time. The advantages of IoT in surveillance extend beyond basic functionalities, offering a range of benefits such as Fast Operation, Automation and Control, Easy Access to Information, and Saving Money that enhance overall security and operational efficiency. Overall, the integration of IoT into surveillance systems provides a more dynamic, efficient, and cost-effective approach to security, enhancing both the functionality and economic viability of these systems. However, the integration of face recognition technology with IoT is essential to significantly enhance the effectiveness of surveillance robots and develop more efficient rapid response systems.

In this paper, a prototype of a cost-effective mobile surveillance robot is proposed. The robot’s intelligent security system is controlled by the Raspberry Pi 4 Model B, known for its advanced computing capabilities. Compared to earlier Raspberry Pi models or other microcontrollers such as the Arduino Uno/Mega, the Raspberry Pi 4 Model B delivers significantly improved processing power. This high-performance processor enhances the robot’s capabilities and overall effectiveness in surveillance tasks.

The main contribution of this work lies in the development and implementation of a cost-effective, intelligent mobile surveillance robot that integrates IoT, face recognition using high-order statistical features, and evidence theory on a Raspberry Pi 4 platform. This system not only enhances real-time monitoring and intruder detection capabilities but also significantly improves recognition accuracy under challenging conditions such as lighting variations, occlusions, and facial expressions. Furthermore, the incorporation of a web-based interface for remote control adds a layer of operational flexibility, making the system suitable for deployment in various industrial and security-sensitive environments.

The main objectives of this research are summarized as follows:

- To design and develop a low-cost intelligent mobile surveillance robot based on Raspberry Pi 4 for real-time security monitoring in industrial and sensitive environments.
- To propose a robust face recognition framework that integrates high-order statistical feature extraction with Dempster–Shafer evidence theory to enhance recognition accuracy under challenging conditions such as illumination variations, facial expressions, and partial occlusions.
- To integrate IoT technology for real-time data transmission, remote monitoring, and alert notification through a web-based interface.
- To evaluate the effectiveness and robustness of the proposed face recognition method using standard benchmark datasets and comparative performance analysis with existing state-of-the-art approaches.
- To validate the practical applicability of the proposed system through experimental implementation and real-time surveillance scenarios.

The proposed surveillance robot is outfitted with a PIR (passive infrared) sensor and a USB camera to detect the presence of intruders within a designated area. By integrating IoT and face recognition technology, the robot can not only monitor the environment but also identify and track individuals, enhancing its ability to provide accurate and timely security responses. This combination of sensors and intelligent technology allows for efficient surveillance, making it a powerful tool in detecting unauthorized access.

When the PIR (passive infrared) sensor detects the presence of a person, the system automatically activates the camera to capture live streaming video and photos. These recordings are then transmitted to the control room through IoT technology, allowing real-time monitoring and analysis of the situation. This automated process ensures that any potential security breach is promptly captured and relayed, enabling quick responses from security personnel.

Face recognition technology based on high-order statistical features and evidence theory is employed to differentiate between known individuals and potential intruders. The proposed method comprises two distinct steps aimed at enhancing recognition accuracy and robustness. Firstly, high-order statistical features are extracted from facial images to capture complex patterns and variations inherent in facial data. Subsequently, evidence theory is employed to integrate these features, providing a comprehensive and reliable basis for face recognition. Hence, this work may be seen as a straightforward additional improvement of the issues proposed by Ben Chaabane et al. [12]. By analyzing and comparing facial features with a pre-existing database, the system can quickly identify authorized personnel while detecting unfamiliar or unauthorized persons. This capability enhances security by allowing for more precise monitoring and reducing false alarms, ensuring that only genuine threats trigger a response.

In addition, when an unknown individual is detected, the system immediately sends an email containing the captured image along with an alert notification to the control room via IoT. Furthermore, a web interface was developed to receive this data and allows for remote control of the robot’s movements over a Wi-Fi connection. This interface provides operators with the ability to monitor the situation in real-time and manually adjust the robot’s position if necessary, enhancing both security and operational flexibility.

The rest of this paper is structured as follows: Section 2 details the proposed robot surveillance system. Section 3 discusses the experimental results, and Section 4 provides the conclusion.

## 2. The Proposed Robot Surveillance System

The proposed surveillance system is designed to carry out high-risk tasks in environments where it would be unsafe or impractical for humans to operate. By leveraging advanced technology, such as automated drones, AI-powered cameras, or remote-controlled robots, the system can monitor and respond to potential threats or hazardous situations in real-time, making it ideal for use in environments like disaster zones, conflict areas, or high-security facilities. This reduces the need for human involvement in potentially life-threatening conditions, while ensuring continuous and efficient surveillance.

In this context, we propose a prototype of a low-cost mobile surveillance robot based on the Raspberry PI 4, which can be integrated into any industrial environment. This intelligent robot detects the presence of intruders using IoT and face recognition technology, providing a reliable and automated security solution. The Unified System architecture and workflow of the surveillance robot is shown in Figure 1.

The system is equipped with a passive infrared (PIR) sensor and a camera, used to capture real-time streaming video and images. These recordings are instantly transmitted to the control room through an IoT network for continuous monitoring.

When the PIR sensor detects the presence of a person, regardless of whether the individual is recognized or registered in the system’s database, the robot automatically activates live-streaming mode and begins capturing photos. This allows for immediate visual data collection, enabling swift response and detailed analysis of potential security breaches or intrusions.

Using the proposed face recognition algorithm, the system can accurately analyze and identify faces by comparing the captured images with the facial data stored in its database. If the facial features match a stored profile, the system recognizes the person as authorized or familiar. If no match is found, the system classifies the individual as unknown or potentially unauthorized. This real-time comparison enables the system to quickly distinguish between familiar faces and possible intruders, improving the overall security and response time of the surveillance system.

Additionally, the system can log interactions with both known and unknown individuals, providing a historical record for future reference or investigation. If a person is detected as an intruder, the system instantly sends a notification to the control room, alerting security personnel of the potential breach. Additionally, the system can send an email with the intruder’s photo attached, providing visual evidence for further action. In cases where the detected individual is a known person, the system will include their name in the notification, ensuring clarity and a quick response. This dual alert mechanism, both real-time notification and email, help streamline the process of identifying and addressing unauthorized access.

Additionally, a web application was developed to allow remote control of the robot, enabling it to approach individuals for better visibility and capture high-quality pictures and videos. Through this application, operators can maneuver the robot to obtain a closer view or focus on specific areas of interest. It is also worth noting that the robot has the flexibility to function as a stationary surveillance system if needed, offering both mobile and fixed monitoring capabilities. This versatility enhances the robot’s effectiveness in various surveillance scenarios, ensuring comprehensive coverage of the area.

### 2.1. Face Detection by Dlib

There are two widely used methods for face detection. The first utilizes the Haar-like feature combined with an AdaBoost classifier, proposed by Viola and Jones [13]. The second method is based on the HOG (Histogram of Oriented Gradients) and SVM (Support Vector Machine) classifiers, introduced by Dalal and Triggs [14].

In the literature [15], a comparison of face detection results is presented between the Viola–Jones algorithm implemented using OpenCV [16] and the HOG algorithm implemented with Dlib [17].

The results demonstrate that for frontal face detection, the performance of the Dlib algorithm significantly outperforms the OpenCV implementation of the Viola–Jones algorithm. Dlib’s use of HOG features and an SVM classifier provides greater accuracy and reliability in detecting frontal faces compared to OpenCV’s traditional Haar-like features and the AdaBoost classifier. This makes Dlib more effective for precise frontal face recognition tasks. For side face detection, while both Dlib and OpenCV have limited capabilities and exhibit poor overall performance, the Dlib algorithm still produces superior detection results compared to OpenCV. Despite the inherent challenges of accurately detecting side faces, Dlib’s HOG and SVM-based approach manages to outperform OpenCV’s Viola–Jones algorithm, showing better detection accuracy in these situations. Based on the comparison outlined above, the Dlib algorithm is chosen for implementation. Its superior performance, particularly in detecting frontal faces, and its ability to outperform the OpenCV Viola-Jones algorithm in both frontal and side face detection make it a more effective solution for this application.

**Figure 2:**
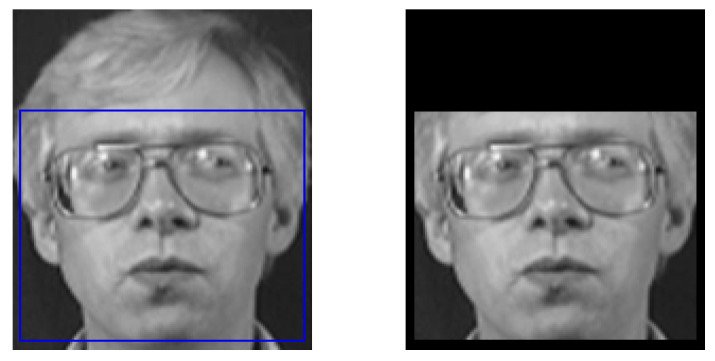
Example of face detection and isolation by Dlib.

Therefore, Dlin’s face detection algorithm is adopted for enhanced accuracy and reliability. The images in the ORL dataset [18] are tested, and an illustrative example of face detection and isolation is presented in Figure 2. The blue frame represents the face bounding box generated by the Dlib face detection algorithm. It indicates the region of interest (ROI) automatically identified as a human face, which is subsequently isolated and used for further processing steps such as alignment, feature extraction, and recognition.

Among the total 400 images in the dataset, two facial images were unable to be detected by face detection algorithms, resulting in the successful face detection of 398 images. The subsequent experimental results and analyses are based exclusively on these 398 successfully detected images. This indicates a high level of accuracy and reliability in the face detection process, allowing for a robust evaluation of the performance of the employed algorithm. After face detection, the face recognition algorithm is implemented to distinguish between company personnel and intruders.

### 2.2. Face Recognition with High-Order Statistical Features and Evidence Theory

Face recognition involves identifying individuals based on their facial features. It encompasses two distinct approaches: biometric identification, which utilizes physical traits for recognition, and behavioral analysis, which assesses actions to verify identity claims. Although both methods have historical use, contemporary practices predominantly favor biometric identification for facial recognition purposes.

Facial recognition is a significant challenge due to the vast volumes of available datasets and various complicating factors. These include facial expressions, lighting conditions, and the presence of accessories like glasses or scarves, which can lead to partial occlusion, further complicating the process.

The objective of a face recognition algorithm is to determine whether two faces are of the same individual or not [19]. Typically, such algorithms consist of three key stages: pre-processing, feature representation, and classifier training. During pre-processing, input images are standardized, noise is eliminated, and essential operations are carried out. The feature representation stage involves extracting relevant features from the images, while the classifier training stage entails training a model based on these extracted features.

The face recognition method proposed in this paper is conceptually different and explores new strategies. The proposed method does not rely on established methods but rather investigates the benefits of integrating various approaches. The proposed method for face recognition is based on high-order statistical features and evidence theory [20]. In the first phase, the statistical features selection approach is applied to each image in order to select the features based on relevance and redundancy characteristics and to construct the attribute images, whereas the evidence theory is used to merge the different information sources. By integrating multiple information sources, each with its reliability, the proposed method can make more informed decisions in face identification tasks.

**Figure 3:**
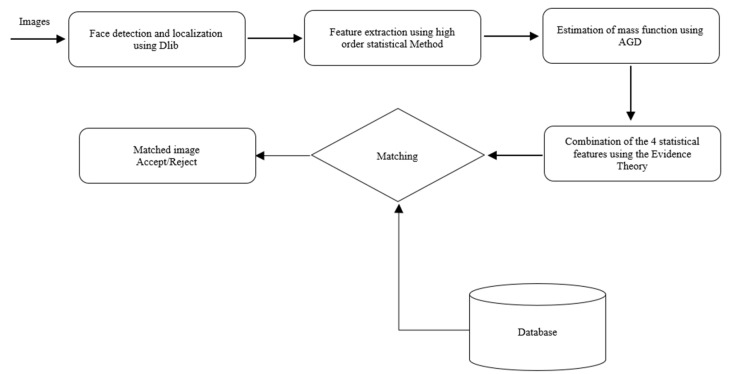
The flowchart of the face recognition method.

Significantly, the proposed face recognition method is structured into two phases. Initially, statistical features are derived from the original images to generate a new image termed as the attribute image [21]. Subsequently, during the second phase, the estimation of the mass functions algorithm [22] and the application of evidence combination and decision rules [23,24,25] are employed to obtain face recognition results. The main components of the proposed face recognition system are depicted in the diagram illustrated in Figure 3.

#### 2.2.1. Feature Extraction

The process of feature extraction from face images is performed using the statistical method. These features capture complex patterns and relationships within the image data that may not be apparent with traditional methods. The idea is to replace the image by the feature extracted from the co-occurrence matrix. The co-occurrence matrix is a common tool in image processing and natural language processing to capture the spatial or relational information between elements in an image or text.

In this work, gray-level co-occurrence matrices (GLCMs) are constructed to capture the spatial relationships between pixel intensities in facial images. The GLCM is computed using a pixel distance of d=1, which captures local texture information that is particularly relevant for facial feature representation. To ensure directional robustness, the GLCM is calculated for four standard orientations, 0°,45°,90°,and 135°, corresponding to horizontal, diagonal, vertical, and anti-diagonal pixel relationships, respectively. The statistical features are extracted independently from each directional GLCM and then averaged to obtain rotation-invariant feature representations. Prior to feature extraction, each GLCM is normalized by dividing all matrix elements by the total number of co-occurring pixel pairs, ensuring that the resulting matrix represents a valid probability distribution. This normalization step improves numerical stability and ensures consistency across images with varying intensity distributions. The selected GLCM parameters provide a balanced trade-off between computational efficiency and feature discriminability, making them suitable for real-time implementation on a resource-limited platform such as the Raspberry Pi 4.

From this matrix, various statistical features can be extracted to characterize the relationships between elements. Some common statistical features include:

Mean: The average value of all the elements in the matrix. It provides a measure of the central tendency of co-occurrence relationships.

(1) $$Mean=\frac{1}{N\times N}\sum_{i=1}^{N}\sum_{j=1}^{N}Cooc(i,j)$$

Variance: Measures the dispersion of values around the mean. Higher variance indicates greater variability in the relationships between elements.

(2) $$Var=\frac{1}{N\times N}\sum_{i=1}^{N}\sum_{j=1}^{N}{\left(Cooc\left(i,j\right)-Mean\right)}^{2}$$

where (N×N) and Cooc(i,j) are respectively the size of the co-occurrence matrix and the value at the ith position in the co-occurrence matrix.

Standard Deviation: The square root of the variance. It quantifies the amount of variation or dispersion present in the co-occurrence relationships.

Entropy: It represents the amount of information or uncertainty in the co-occurrence matrix. Higher entropy indicates greater randomness or disorder in the relationships between elements.

(3) $$Entrop=-\sum_{i=1}^{N}\sum_{j=1}^{N}Cooc\left(i,j\right).log(Cooc\left(i,j\right))$$

Log denotes the natural logarithm.

Energy: Also known as uniformity or angular second moment, it measures the sum of squared elements in the co-occurrence matrix. High energy indicates a high degree of homogeneity in relationships.

(4) $$Energ=\frac{1}{N\times N}\sum_{i=1}^{N}\sum_{j=1}^{N}{\left(Cooc\left(i,j\right)\right)}^{2}$$

where

$Cooc\left(i,j\right)$ denotes the normalized gray-level co-occurrence matrix (GLCM) value corresponding to pixel intensity levels i and j.

i,j∈{1,2,…,N}, where N represents the number of gray levels in the quantized image; N×N is the size of the GLCM; and values where Cooc(i,j)=0 are excluded from the summation to avoid numerical instability.

Contrast: Measures the intensity contrast between neighboring elements in the co-occurrence matrix. High contrast values indicate large intensity differences, while low contrast values indicate similar intensities.

Correlation: Measures the linear dependency between pairs of elements in the co-occurrence matrix. It indicates how much one element is related to another. Positive values denote positively correlated elements, negative values denote negatively correlated elements, and zero indicates no correlation.

Homogeneity: It reflects the closeness of the distribution of elements in the co-occurrence matrix to the diagonal. Higher homogeneity values indicate that the elements are concentrated along the diagonal, implying a high degree of uniformity in relationships.

These are just a few examples of statistical features that can be derived from co-occurrence matrices. The choice of features depends on the specific application and the desired characterization of the relationships between elements. In this work, the features selection is based on their relevance and redundancy characteristics. Features that are relevant for distinguishing between different faces are identified and retained, while redundant or less informative features are discarded. This helps in reducing the dimensionality of the feature space and focusing on the most discriminative features. These statistical features serve as the basis for further analysis and processing in the face recognition task.

#### 2.2.2. Use the Evidence Theory for Classification

The purpose of classification is to classify the images into different classes. The idea of using Dempster–Shafer (DS) evidence theory for image classification is to integrate the information from multiple sources while considering the uncertainty associated with each piece of evidence. Initially, features are extracted from the input images using statistical methods. Each feature highlights specific characteristics of the original image. The DS evidence theory is then used to fuse the features extracted from the co-occurrence matrix. In DS theory, each piece of evidence (feature) is represented by a belief function, which assigns degrees of belief to different hypotheses (classes, i.e., faces). Finally, based on the combined belief function, a classification decision is made. This decision could involve assigning the image to one or more predefined classes, depending on the evidence provided by the features extracted from the co-occurrence matrix.

Dempster–Shafer theory (DS) [26,27], also known as the theory of belief functions, provides a framework for reasoning with uncertain or incomplete information. Unlike traditional probability theory, DS theory allows for the representation of uncertainty more flexibly by assigning degrees of belief to sets rather than just single events.

In the present study, the frame of discernment Ω composed of n single mutually exclusive subsets $H_{n}$ contains clusters or faces ($C_{i}$), which are symbolized by:

(5) $$\Omega =\left\{\left.H_{1},H_{2},\ldots ,H_{n}\right\}=\right.\left\{\left.C_{i}\right\}\right.$$

where 1≤i≤n.

In order to express a degree of confidence for each proposition A of $2^{\Omega }$, it is possible to associate an elementary mass function m(A), which indicates the degree of confidence that one can give to this proposition. Formally, this description of m can be represented with the following three equations:

(6) $$\left\{\begin{array}{c} m:\;2^{\Omega }\to \left[0,1\right]\\ m\left(\emptyset \right)=0\;\;\;\;\;\;\;\;\;\\ \sum_{A\subseteq \Omega }m\left(A\right)=1 \end{array}\right.$$

where

Ω is the frame of discernment containing all possible face classes;

$C_{i}$ denotes the ith face class (identity), with i = 1, 2, …, n;

$2^{\Omega }$ represents the power set of Ω;

m(A) is the basic probability assignment (mass function) associated with hypothesis A⊆Ω;

m(∅)=0 ensures that no belief is assigned to the empty set;

$\sum_{A\subseteq \Omega }m\left(A\right)=1$ guarantees normalization of belief masses.

The purpose of segmentation is to divide the image into uniform regions. The idea of using the evidence theory for image segmentation is to fuse one by one the features coming from the co-occurrence matrix of each image. The Gaussian distribution model [28] is applied to the statistical features to be combined. Then, the mass functions are combined using the Dempster–Shafer (DS) combination theory to obtain the final segmentation results.

Masses of simple hypotheses $C_{i}$ are derived based on the assumption that the attributes x for the class i follow a Gaussian distribution. For each hypothesis $C_{i}$, the probability density function (PDF) of the Gaussian distribution is used to describe the probability that a data point belongs to the class i. The formula for the mass function based on the Gaussian distribution for the class i is as follows:

(7) $$m\left(C_{i}\right)=\frac{1}{\sqrt{2\pi \sigma_{i}^{2}}}exp(-\frac{{\left(x-\mu_{i}\right)}^{2}}{2\sigma_{i}^{2}})$$

where

$m\left(C_{i}\right)$ is the mass of hypothesis $C_{i}$, representing the likelihood that the attribute belongs to class i. x is the attribute value being evaluated. $\mu_{i}$ is the mean of the Gaussian distribution for class i. $\sigma_{i}^{2}$ is the variance of the Gaussian distribution for class i.

The mass function assigned to double hypotheses (i.e., when there is uncertainty between two possible clusters) depends on the mass functions of each individual hypothesis. In the context of evidence theory, such as Dempster–Shafer theory, the mass function for a double hypothesis is derived by combining the masses of the individual hypotheses while accounting for the uncertainty between them.

Let us denote two simple hypotheses as $C_{i}$ and $C_{j}$. The mass function for the double hypothesis $C_{i}\cup C_{j}$, which represents the uncertainty that the attribute could belong to either class i or class j, can be expressed as:

(8) $$m\left(C_{i}\cup C_{j}\right)=\frac{1}{\sqrt{2\pi \sigma_{ij}^{2}}}exp(-\frac{{\left(x-\mu_{ij}\right)}^{2}}{2\sigma_{ij}^{2}})$$

where

(9) $$\left\{\begin{array}{c} \mu_{ij}=(\mu_{i}+\mu_{j})∕2\\ \sigma_{ij}=max(\sigma_{i}+\sigma_{j}) \end{array}\right.$$

x denotes the observed feature value extracted from the test image;

$\mu_{i}$ and $\sigma_{i}^{2}$ represent the mean and variance of the Gaussian distribution associated with class $C_{i}$, estimated from training samples.

Once the mass functions of the four attributes are estimated, their combination can be performed using Dempster’s rule of combination (also known as the orthogonal sum) from Dempster–Shafer theory. This rule is used to combine evidence from multiple sources to estimate the final belief or mass function. The orthogonal sum for combining two mass functions $m_{1}$ and $m_{2}$ is represented as follows:

(10) $$m_{12}\left(C_{i}\right)=\frac{1}{1-K}\sum_{A_{1}\cap A_{2}=C_{i}}m_{1}\left(A_{1}\right).m_{2}\left(A_{2}\right)$$

where $m_{12}\left(C_{i}\right)$ denotes the combined mass assigned to hypothesis $C_{i}$, where the summation is performed over all focal elements $A_{1}$ and $A_{2}$ such that their intersection equals $C_{i}$. This formulation follows the classical Dempster–Shafer orthogonal sum rule for combining independent evidence sources. K is the conflict term, which quantifies the conflict between the two mass functions and is given by:

(11) $$K=\sum_{A_{1}\cap A_{2}=\emptyset }m_{1}\left(A_{1}\right).m_{2}\left(A_{2}\right)$$

The conflict term K quantifies the degree of contradiction between evidence sources and is computed by summing the products of mass functions whose corresponding focal elements have an empty intersection. A higher value of KKK indicates greater disagreement between sources of evidence.

In the DS combination theory [29], the mass functions of the four attributes are fused together to generate a single value. After calculating the orthogonal sum of the mass functions for the four attributes, the decisional procedure for classification consists of choosing one of the most likely hypotheses $C_{i}$. This is typically done by evaluating the final mass functions and identifying the hypothesis with the highest mass (i.e., the most supported hypothesis based on the combined evidence).

## 3. Experimental Results and Discussion

### 3.1. Design, Implementation, and Experimental Evaluation of the Proposed Mobile Surveillance Robot with Face Recognition

**Figure 4:**
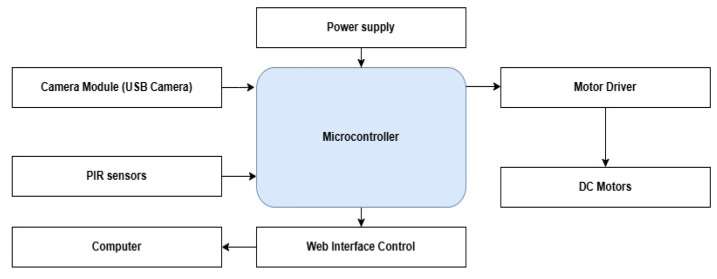
Block diagram of the robot system.

The electrical design of the robot is outlined, with a schematic diagram of the proposed system created using Fritzing software (Version 1.0.6). The diagram of the robot system is given by the following Figure 4, which incorporates the following components:

- Four Motors: Responsible for movement, controlled with the information transmitted by the sensors.
- Dual H-Bridge Motor Driver L298N: Typically used to control motor speed and rotation direction.
- PIR Sensors: Used to detect human motion with a sensitivity range of about 7 m.

- Raspberry Pi4: Used to control the proposed intelligent security system.
- USB camera: Used to stream live video to a user at a remote location, enabling real-time monitoring. Additionally, the camera captures images of an intruder whenever motion is detected, enhancing the system’s security functionality.
- Power Supply: Provides the necessary voltage and current to power the system. The motors are powered by a 12 V battery, which delivers the necessary voltage to the motors through a motor driver, ensuring proper regulation and control of power. Additionally, a power bank is utilized to supply power to the Raspberry Pi, providing a stable and portable energy source for the microcontroller, allowing it to manage the system’s operations independently of the motor power supply.
- Wiring and Connectors: Used to connect all components and ensure communication and power distribution.

**Figure 5:**
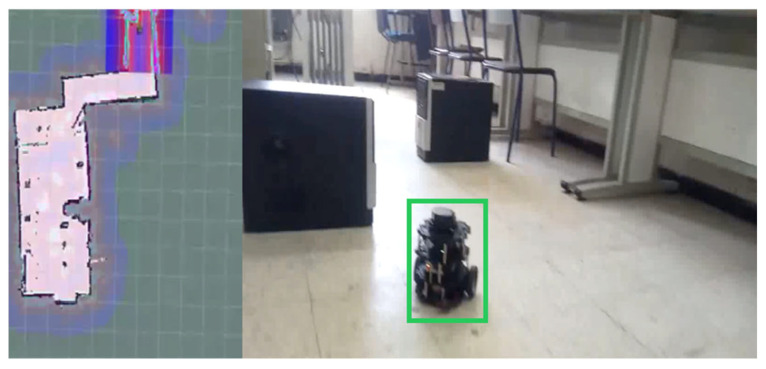
The prototype of the robot.

This schematic offers a clear visualization of how these components are integrated into the overall electrical system of the robot. The prototype of the surveillance robot is presented in Figure 5. The green frame highlights the detected robot and indicates the region of interest (ROI) used for tracking and navigation purposes. The colored areas on the left represent the environmental mapping and obstacle detection results, where different colors visualize spatial information generated by the robot’s sensing and localization system. These color variations correspond to mapped regions, detected obstacles, and free navigation space used to support autonomous movement and decision-making.

**Figure 6:**
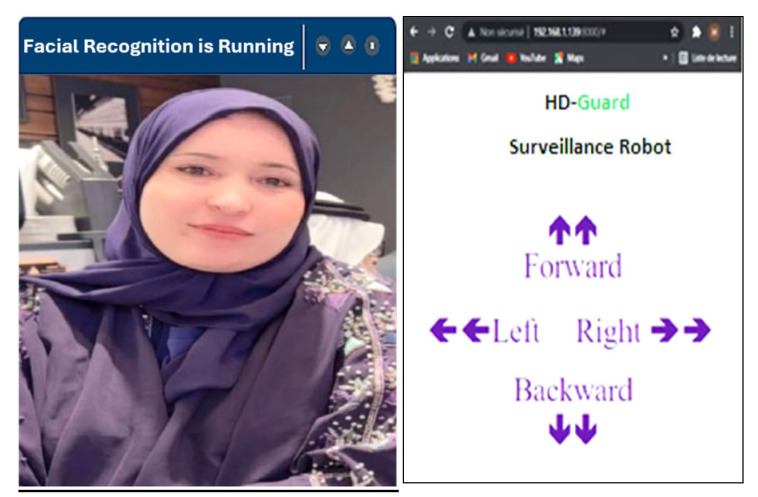
Web interface and face recognition.

When the PIR (passive infrared) sensor detects human motion, the system activates a live video stream to monitor the scene. As the video stream starts, face recognition software is employed to analyze and verify the presence of any personal facial data. If the system identifies the individual as someone stored in its database, it cross-references the data and displays the person’s name on the screen. Figure 6 represents the scenario in which a known individual is detected by the system. When the person is recognized, the system identifies them and displays their name on the screen.

**Figure 7:**
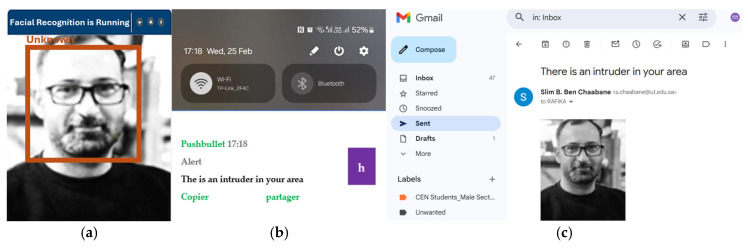
(**a**) An intruder detected by the system, (**b**) an alert notification, (**c**) email containing the person’s photo sent when the system detects an intruder.

If the facial data of the detected individual does not match any entries in the system’s database, the monitoring system will label the person as “unknown,” as demonstrated in Figure 7a. Simultaneously, the system triggers an alert by sending a notification and an email containing a captured image of the individual to the room control center, as illustrated in Figure 7b and Figure 7c, respectively. This ensures timely awareness and response to potential security concerns.

In order to evaluate the performance of the proposed face recognition method, we conducted different experiments using the same dataset. Firstly, we compared the accuracy of face recognition methods using different feature extraction techniques. Secondly, we evaluate the performance of face recognition algorithms using different classifiers: Support Vector Machines (SVMs) [12,19], k-Nearest Neighbors (KNNs) [20], and K-means clustering [30]. In addition, we compared these results to those obtained by the state-of-the-art face recognition methods such as the DCP and LBP method [31], the facial recognition system using local binary patterns (LBP) [32], and the face recognition method based on statistical features and the SVM classifier [12].

**Figure 8:**
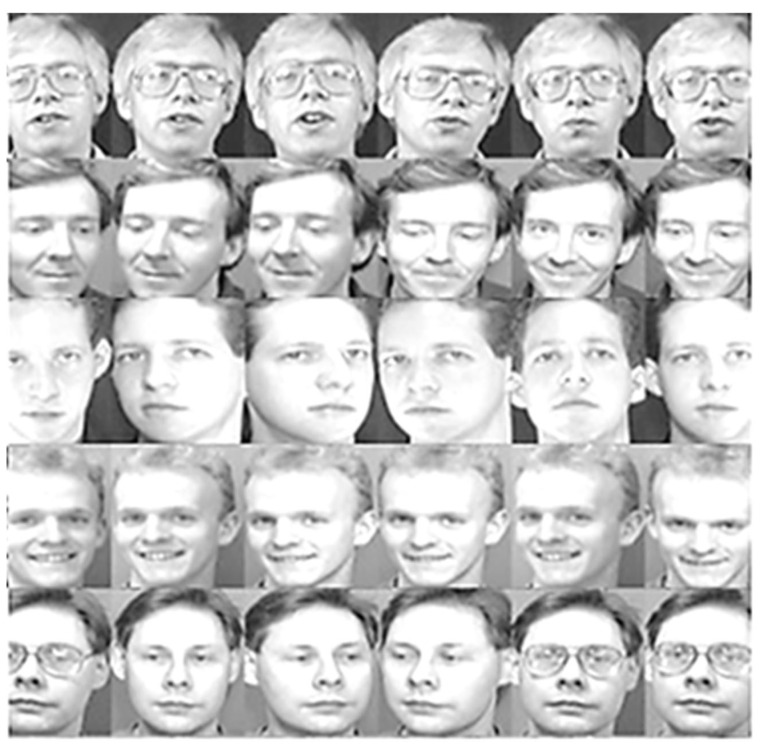
Samples of the ORL face images.

The ORL dataset consists of 400 images taken from 40 different people, with 10 poses for each person. The images were then converted into JPEG image format without changing their size. The images are stored in gray-level format, are 8 bits, and have an intensity range from 0 to 255. Five individuals with six images for each person of the ORL face images are shown in Figure 8.

The images were then split into two sets. One set was used for training, and the other set was used for testing. Frequently, datasets are isolated into training and testing datasets in the proportion of 45:55. Hence, 180 images are randomly chosen as training sets with 220 images from all cases. In order to reduce the training set dimension, the training sets include 30, 60, 90, 120, 150, and 180 images according to the chosen pose count. For each person, poses with the same indices are chosen for the corresponding set.

**Figure 9:**
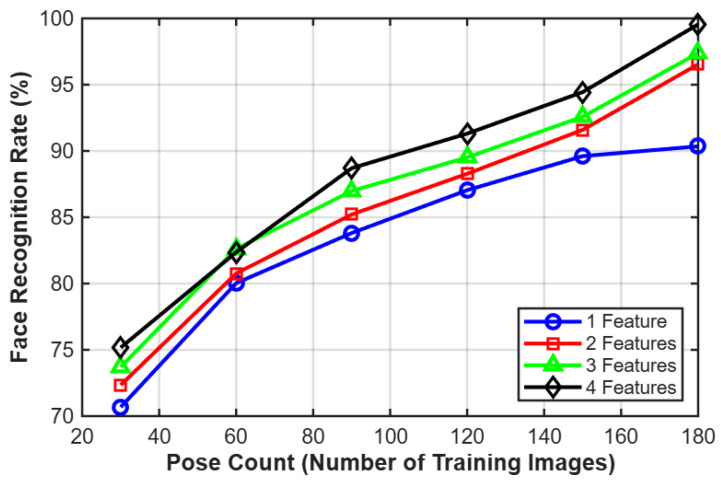
Face recognition rates at different pose counts.

Table 1 shows the results obtained by the proposed method according to increasing pose count and the dimension of feature vectors. In addition, Figure 9 shows the face recognition rates of our model at different pose counts. The blue, red, green, and black lines represent the face recognition rates by using one, two, three, and four features, respectively.

We can notice that, as we increase the pose count, the accuracy of the model increases. At pose count 30 and using only one feature, the accuracy was around 75.1743%. At pose count 150 and using four features, the accuracy increased to about 94.435% and then continued to rise until pose count 180 with four features where a peak level (99.5454%) in recognition performance is obtained with the proposed method.

In our experiments, 180 images are randomly chosen as training sets for each class. To ensure statistical reliability, each experiment reported in Table 1 is repeated 10 times with different random selections of training images. The results are presented as the mean recognition rates along with their standard deviations, providing a more robust assessment of the system’s performance.

To evaluate our method, we compare and contrast the results of the proposed method with other published reports recently applied to face recognition. These include the DCP and LBP method [31], the facial recognition system using local binary patterns (LBP) [32], and the face recognition method based on statistical features and SVM classifier [12]. Table 2 shows the results obtained from a previous study [12,31,32] on the ORL database using 45% of each individual’s samples in the training set and 55% of the samples in the test set. From Table 2, we notice that a peak level (99.5454%) of the face recognition rate and true positive (219 images) are obtained with the proposed method. Experimental comparisons use different evaluation protocols, including the face recognition rate (FRR) and equal error rate (EER) [33]. The face recognition rate (FRR) is defined as the percentage of correctly identified faces out of total number of faces. FRR is calculated using the following formula:

(12) $$FRR(\%)=\frac{TP}{TP+FN}\times 100$$

where FRR is the face recognition rate; TP represents true positive, i.e., the number of images where the face was detected; FN represents false negative, i.e., those images where the face was not detected.

The equal error rate (EER) is the minimum probability of making a mistake while recognizing a person’s identity. In order to calculate EER, we need two parameters: (1) the False Acceptance Rate (FAR), which is the ratio of incorrectly accepted images to the total number of images where the faces were detected; (2) the True Rejection Rate (TRR), which is the ratio between the number of incorrect rejected images to the total number where the faces were not detected.

**Figure 10:**
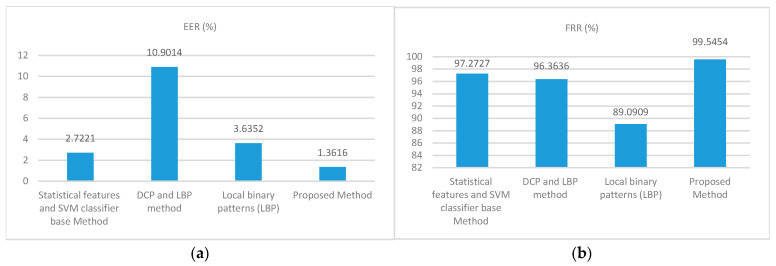
The recognition performance of different approaches on ORL face images, (**a**) EER, (**b**) FRR.

Table 3 shows the EER numerical comparison of face recognition based on statistical features and SVM classifier (SFSVM) [12], DCP and LBP method [31], and local binary patterns (LBP) [32]. The intuitive comparisons in terms of EER and FRR between these methods are plotted in Figure 10.

From Table 3 and Figure 10, we can see that the proposed method clearly outperforms the other approaches with an EER of 1.3616%, and an FRR of 99.5454%. Hence, higher accuracy rates are obtained with the proposed method. The experiments showed that the proposed method had a lower error rate than the other methods [12,31,32].

The proposed method was tested on a variety of images, demonstrating robustness and high accuracy rates even with poor-quality inputs. Our experimental results revealed that the choice of feature extraction technique significantly impacted face recognition performance. Specifically, the highest accuracy was achieved when the statistical feature extraction method was employed. Furthermore, the face recognition performance improved notably when the classification process utilized evidence theory. Overall, the proposed method proves effective and can be applied to face recognition tasks and mobile.

### 3.2. System Risks and Limitations

Despite the promising performance achieved by the proposed mobile surveillance robot, several system risks and operational limitations must be considered when deploying the system in real-world environments. The face recognition module relies on visible-light images captured by a standard USB camera; therefore, in low-light or nighttime conditions, image quality may degrade, leading to reduced face detection and recognition accuracy. This issue could be mitigated in future work by integrating infrared (IR) cameras, low-light image enhancement techniques, or adaptive illumination modules. In addition, the system depends on IoT and Wi-Fi connectivity to transmit video streams, alerts, and images to the control room. Network congestion or limited bandwidth may introduce communication delays, potentially affecting real-time monitoring and response. The impact of such delays could be reduced through the use of edge computing strategies, local data buffering, or adaptive data compression techniques.

Another limitation is related to the PIR sensor, which may generate false alarms due to its sensitivity to sudden temperature changes and environmental factors such as airflow or nearby heat sources. Although PIR sensors are cost-effective and efficient for motion detection, system reliability could be improved by fusing data from additional sensors, such as ultrasonic sensors or vision-based motion detection methods. Furthermore, camera stability during robot movement represents a challenge, as vibrations and abrupt motions can cause motion blur or unstable video capture, negatively affecting face recognition accuracy. This issue may be addressed by incorporating mechanical stabilization mechanisms, software-based image stabilization, or by temporarily pausing the robot during face acquisition. Finally, scalability and computational constraints must be considered; although the Raspberry Pi 4 provides adequate processing power for the proposed system, executing multiple tasks simultaneously, such as video streaming, face recognition, and IoT communication, may increase latency under heavy workloads. Future enhancements could focus on algorithm optimization or the use of hardware acceleration to improve overall scalability and system performance.

Although the experimental evaluation in this study is conducted on the ORL face dataset, which is a widely used benchmark in face recognition research, it should be noted that ORL represents a relatively constrained environment with controlled background and limited variations in pose and illumination. In contrast, unconstrained datasets typically involve significant challenges such as complex backgrounds, uncontrolled lighting conditions, spontaneous facial expressions, occlusions, and image quality degradation. The proposed face recognition framework is inherently designed to handle such variability through the use of high-order statistical features, which capture robust texture and structural information, and through Dempster–Shafer evidence theory, which explicitly models uncertainty and fuses complementary information sources. These characteristics are expected to improve generalization when applied to unconstrained scenarios. However, a comprehensive quantitative evaluation on large-scale unconstrained datasets such as Labeled Faces in the Wild (LFW) or FERET is beyond the scope of the current work and will be addressed in future studies. Such evaluations will further validate the robustness, scalability, and real-world applicability of the proposed method under highly unconstrained conditions.

In addition, the detection and recognition of masked faces constitute a critical challenge for modern surveillance systems due to the partial occlusion of discriminative facial regions such as the nose and mouth. Although the experimental evaluation in this work does not explicitly include masked face datasets, the proposed face recognition framework is inherently more robust to partial occlusions compared to holistic appearance-based methods. This robustness stems from the use of high-order statistical features extracted from local texture patterns, which primarily rely on the eye, forehead, and periocular regions that often remain visible when face masks are worn. Furthermore, the integration of Dempster–Shafer evidence theory enables the fusion of multiple feature sources while explicitly modeling uncertainty, thereby reducing the negative impact of missing or unreliable information caused by occlusions. Nevertheless, it is expected that recognition accuracy may decrease as the degree of occlusion increases. A comprehensive quantitative evaluation on publicly available masked face datasets, such as RMFD or MAFA, is planned as future work to further validate the effectiveness and adaptability of the proposed approach in real-world masked face surveillance scenarios.

### 3.3. Computational Complexity Analysis

Since the proposed surveillance system is deployed on a resource-limited Raspberry Pi 4 platform, analyzing the computational complexity of the main processing stages is crucial to assess real-time feasibility. Let an input face image have a size of (N×N) pixels, and let F denote the number of extracted statistical features per image.

- High-order statistical feature extraction:
  The computation of the gray-level co-occurrence matrix (GLCM) requires scanning the image once, leading to a time complexity of $O(N^{2})$. The extraction of statistical features (mean, variance, entropy, energy, etc.) from the GLCM involves simple arithmetic operations over the matrix, resulting in a complexity of O(F). Therefore, the overall complexity of the feature extraction stage is $O(N^{2}+F)$.
- Formation of attribute images:
  Attribute images are constructed by mapping the extracted statistical features to image representations. This process involves pixel-wise operations over the original image, resulting in a computational complexity of $O\left(N^{2}\right)$ per attribute image. Since the number of attributes is limited and fixed, this stage remains computationally efficient.
- Evidence theory-based fusion:
  The application of Dempster–Shafer evidence theory involves computing mass functions for each feature and combining them using Dempster’s rule. For CCC classes and FFF features, the complexity of mass function estimation is $O\left(F.C\right).$ The fusion of evidence using Dempster’s combination rule has a complexity of $O\left(C^{2}\right),$ which remains manageable due to the limited number of classes in practical scenarios.
- Classification process:
  The final classification step consists of selecting the hypothesis with the maximum combined belief value, which requires a linear search over the C classes. Thus, the classification complexity is O(C).
- Overall complexity:
  Combining all stages, the overall computational complexity of the proposed face recognition pipeline is dominated by the image-level operations and can be approximated as $O\left(N^{2}+C^{2}\right)$ This level of complexity is suitable for real-time implementation on the Raspberry Pi 4, as confirmed by the experimental evaluation and stable system performance during deployment.

## 4. Conclusions

In this paper, a mobile surveillance robot based on Raspberry Pi 4 and IoT is presented. The system can be operated remotely through a web interface and Wi-Fi connection. The robot is designed to continuously monitor a specific area and provides live video streaming when it detects human movement. A new method for face recognition based on high-order statistical features and evidence theory is used to identify whether the detected person is familiar or unfamiliar. If the system identifies an intruder person, it will send out alert notifications and emails via IoT.

The performance of the proposed human face recognition method is evaluated and a comparative analysis with existing techniques is conducted to demonstrate the effectiveness and robustness of the face recognition algorithm used in this framework. We obtained the highest recognition rate as 99.5454% with the proposed method. Considering the weighted averages of the recognition rates, the proposed recognition method gave better results compared to some existing approaches. Due to these features, the designed robot is suitable for surveillance applications in various environments.

This work can be expanded to develop a mobile security robot able to move autonomously, avoid obstacles, and select the most efficient trajectory. Additionally, more advanced face recognition algorithms could be implemented, allowing the robot to also recognize vehicle license plates.

## Figures and Tables

**Table 1:** Face recognition rates of the proposed method according to increasing pose count and the dimension of feature vectors.

Dimensions of Feature Vectors/ Pose Count Per Individual in Training	1 (30 Training Images)	2 (60 Training Images)	3 (90 Training Images)	4 (120 Training Images)	5 (150 Training Images)	6 (180 Training Images)
1	70.68 ± 0.85	80.03 ± 0.92	83.81 ± 0.88	87.04 ± 0.90	89.61 ± 0.95	90.35 ± 0.87
2	72.34 ± 0.80	80.76 ± 0.88	85.21 ± 0.91	88.28 ± 0.85	91.59 ± 0.89	96.54 ± 0.92
3	73.71 ± 0.82	82.61 ± 0.87	86.98 ± 0.90	89.52 ± 0.88	92.58 ± 0.91	97.38 ± 0.89
4	75.17 ± 0.83	82.36 ± 0.86	88.70 ± 0.92	91.31 ± 0.87	94.44 ± 0.90	99.55 ± 0.85

**Table 2:** Face detection evaluation results.

	Total Faces	DCP and LBP Method [31]	Local Binary Patterns (LBP) [32]	Statistical Features and SVM Classifier Base Method [12]	Proposed Method
True Positive	220	212	196	214	219
False Positive	220	8	24	6	1
False Negative	220	71	42	59	29
Detection Accuracy Rate		96.3636	89.0909	97.2727	99.5454

**Table 3:** The recognition performance of different approaches on Olivetti Research Laboratory (ORL) database.

No	Method	EER (%)	FRR (%)
1	Statistical features and SVM classifier base Method [12]	2.7221	97.2727
2	DCP and LBP method [31]	10.9014	96.3636
3	Local binary patterns (LBP) [32]	3.6352	89.0909
4	Proposed Method	1.3616	99.5454

## Data Availability

The original contributions presented in this study are included in the article. Further inquiries can be directed to the corresponding author.

## Abbreviations

The following abbreviations are used in this manuscript:

AI	Artificial Intelligence
CV	Computer Vision
IoT	Internet of Things
PIR	Passive Infrared Sensor
SVM	Support Vector Machine
HOG	Histogram of Oriented Gradients
LBP	Local Binary Patterns
DCP	Dual Cross Patterns
DS	Dempster–Shafer (Evidence Theory)
PDF	Probability Density Function
FRR	Face Recognition Rate
EER	Equal Error Rate
FAR	False Acceptance Rate
TRR	True Rejection Rate
ORL	Olivetti Research Laboratory (Face Dataset)
FFT	Fast Fourier Transform
JPEG	Joint Photographic Experts Group
Wi-Fi	Wireless Fidelity
